# When One Tap Isn’t Enough: A Case of Pseudotumor Cerebri Requiring Venous Sinus Stenting

**DOI:** 10.7759/cureus.94778

**Published:** 2025-10-17

**Authors:** Sherilyn Nguyen, Nicholas Kato, Salomon D Salama

**Affiliations:** 1 Nova Southeastern University Dr. Kiran C. Patel College of Osteopathic Medicine, Nova Southeastern University, Fort Lauderdale, USA; 2 Ophthalmology, Eye Physicians and Surgeons, Boca Raton, USA; 3 Ophthalmology, Baptist Health South Florida, Boca Raton, USA

**Keywords:** cerebral venous sinus stenosis, idiopathic intracranial hypertension, lumbar puncture (lp), pseudotumor cerebri (ptc), venous sinus stent

## Abstract

Pseudotumor cerebri (PTC), or idiopathic intracranial hypertension (IIH), is characterized by elevated intracranial pressure. While most cases are idiopathic, secondary causes, such as cerebral venous sinus stenosis (CVSS), must be thoroughly evaluated, as they require targeted management. Clinical manifestations commonly include headache, pulsatile tinnitus, and transient visual disturbances. If left untreated, severe cases may lead to permanent vision loss due to sustained optic nerve compression. In this report, we present a case of PTC secondary to venous sinus stenosis and review the current approaches to diagnosis and treatment.

## Introduction

Idiopathic intracranial hypertension (IIH), also known as pseudotumor cerebri (PTC), is a neurological condition characterized by elevated intracranial pressure (ICP) without a cerebral mass, hydrocephalus, infection, or hypertensive encephalopathy. Although it can occur in other demographics, it primarily impacts young, obese women of reproductive age [1]. Chronic headaches, transient visual obscurations, pulsatile tinnitus, diplopia, and papilledema are typical clinical findings [2]. If left untreated, patients may develop irreversible vision loss. Elevated ICP leads to papilledema, which distorts vision and, over time, causes optic nerve damage. Vision loss in IIH occurs through two primary mechanisms: ischemia and mechanical compression of the optic nerve [3]. Reduced arterial perfusion and venous stasis compromise the blood supply to the nerve. Notably, the degree of optic disc edema is closely associated with the severity of vision loss [4].

Though the exact cause of IIH is still unknown in many patients, new research points to a possible link to abnormalities of the dural venous sinuses, such as transverse sinus stenosis (TSS). Increased ICP may result from this narrowing's impact on venous outflow obstruction or inadequate absorption of cerebrospinal fluid (CSF). Recognizing TSS as a possible underlying factor in IIH has important diagnostic and therapeutic implications, including the role of venous sinus stenting as a treatment modality in refractory cases.

We present a case of PTC linked to TSS, highlighting clinical findings, diagnostic process, and management challenges. We also discuss the growing knowledge of the pathophysiological relationship between IIH and venous sinus stenosis.

## Case presentation

**Figure 1:**
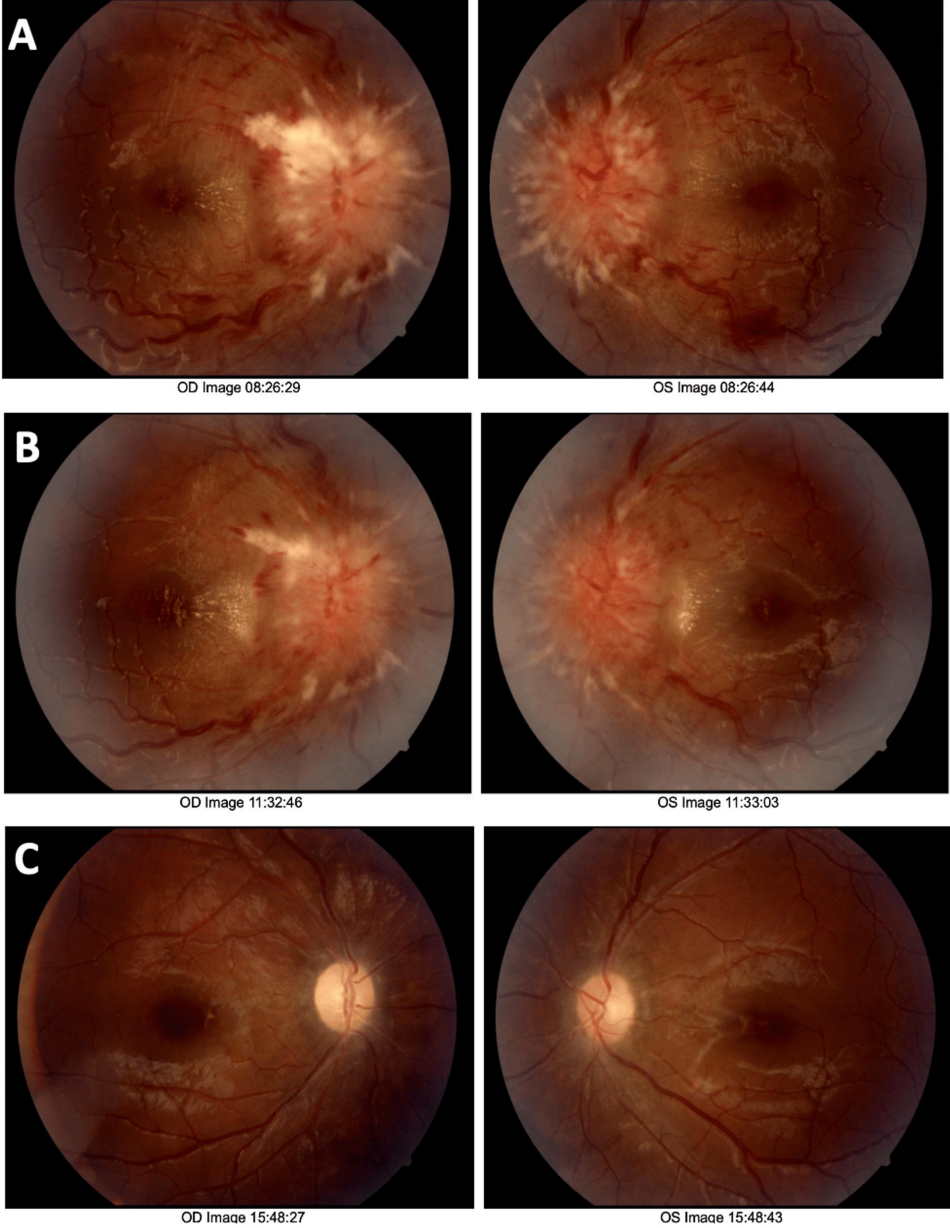
Fundus photography. (A) Initial fundus photograph demonstrates severe papilledema with elevated, blurred optic disc margins and obscuration of vessels on and leaving the optic disc. (B) Two-week follow-up shows minimal improvement in papilledema. There is evidence of reduced hemorrhage; however, disc elevation and blurred margins remain unchanged. (C) Ten-month follow-up demonstrates significant improvement in papilledema, with clear disc margins and resolution of disc elevation. The image shows peripapillary atrophy in both eyes, highlighting residual damage resulting from elevated intracranial pressure.

A 20-year-old obese Hispanic female presented to an outpatient ophthalmology clinic with worsening vision loss and pain in the right eye. Symptoms began one month ago and have progressed over time. She had no past medical history, including diabetes, hypertension, or systemic illness. She reported no use of medications such as oral contraceptive pills, antibiotics, or supplements. She denied weakness, aphasia, or sensory loss. Her body mass index (BMI) was 35.63 kg/m², and her mental status was intact. Visual acuity initially presented as counting fingers at 1 ft. in the right eye and 20/60 in the left eye. Her pupils were reactive, and no afferent pupillary defect was present. Her intraocular pressures were 9 and 12 for the right and left eyes, respectively. Her extraocular muscles were intact, and confrontational visual fields were full bilaterally. Fundus photography revealed severe papilledema with blurred disc margins (Figure 1A), raising concern for PTC.

**Figure 2:**
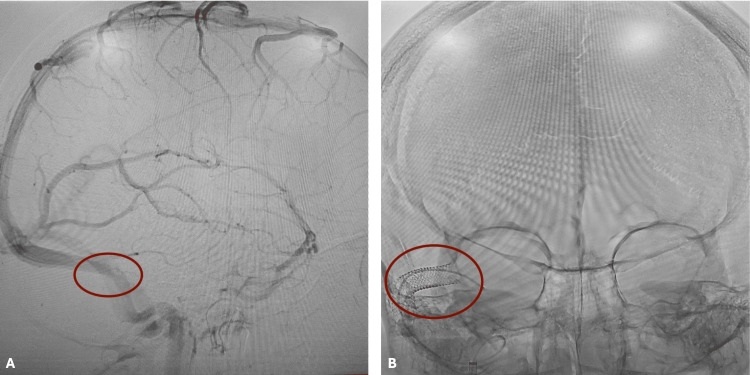
Neuroimaging: (A) preoperative venogram demonstrating transverse venous sinus stenosis (circled in red); (B) postoperative head X-ray showing stent placement (circled in red).

The patient was initially referred to the nearest emergency department; however, she instead presented to a different hospital and received care there. While in the hospital, she underwent a lumbar puncture, which revealed an elevated opening pressure of 35 cm H₂O and 20 cc of clear cerebrospinal fluid. Cytology was not documented; however, the absence of fever, meningismus, and systemic symptoms made an infectious etiology unlikely. Venous magnetic resonance imaging (MRI) was not initially performed due to the patient’s lack of insurance. Following lumbar puncture, her symptoms improved, and she was discharged on 500 mg twice daily of extended-release Diamox (acetazolamide). Two weeks following hospital discharge, she returned to the ophthalmology clinic for recurrent symptoms. Due to insurance limitations at the previous hospital, imaging was arranged pro bono. MRI and MR venography performed the same day demonstrated right TSS (Figure 2A). Intracranial mass and venous thrombosis were excluded, while TSS was confirmed. A repeat lumbar puncture yielded results unchanged from the initial study. Based on these findings, the patient was diagnosed with PTC secondary to TSS and subsequently underwent transverse venous sinus stenting (Figure 2B).

**Figure 3:**
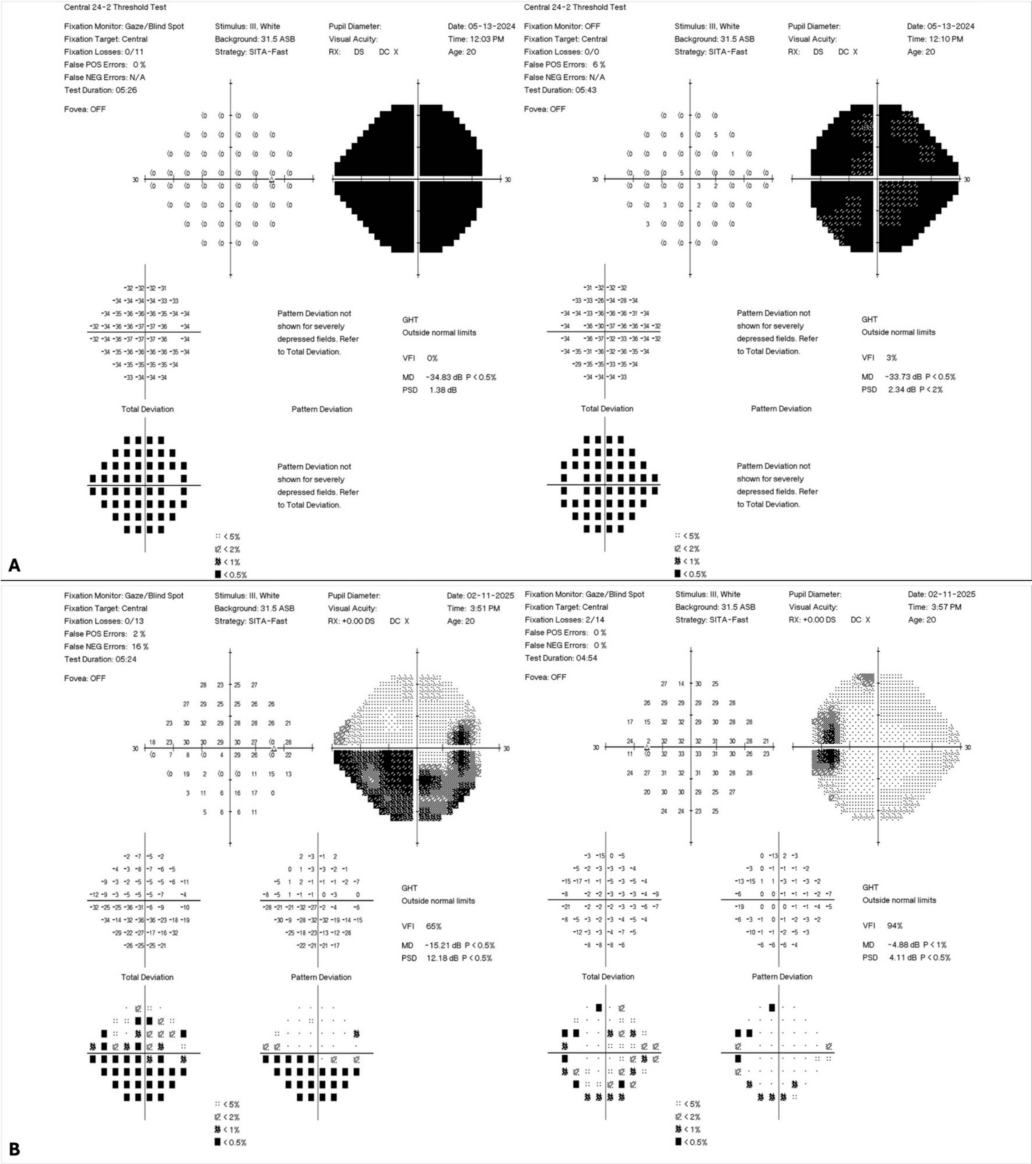
Perimetry test or visual field test. (A) Initial perimetry shows 360 degrees of constriction, demonstrating severe visual field loss in both eyes. (B) Perimetry results at 10-month follow-up show significant improvement in the visual field, more in the left than the right eye. The right eye shows lingering inferior arcuate visual field loss, while the left eye demonstrates scattered defects.

Following stent placement, the patient was placed on dual antiplatelet therapy with 75 mg of Plavix (clopidogrel) and aspirin for several months as prophylaxis against stent thrombosis. At the ten-month follow-up, the patient reported resolution of symptoms, and repeat fundus photography revealed significant improvement in papilledema (Figure 1C). Visual acuity improved to 20/70 and 20/25 in the right and left eyes, respectively. Initial perimetry results showed complete bilateral visual field loss (Figure 3A). Follow-up perimetry demonstrated marked improvement in visual field sensitivity compared to baseline; however, residual deficits were noted, consistent with permanent optic nerve damage (Figure 3B).

## Discussion

IIH is a neurological condition characterized by elevated ICP. Obese women of reproductive age, as seen in this patient, are most often impacted, and symptoms including headache, temporary visual loss, pulsatile tinnitus, diplopia, and papilledema are classic symptoms [5]. Medications and exogenous substances, such as excessive vitamin A intake, tetracyclines, and lithium, can lead to elevated ICP. Continuous compression of the optic nerve due to impaired CSF dynamics can lead to progressive and irreversible vision loss if treatment is not received. Recent research has linked dural venous sinus abnormalities, specifically TSS, as a potential cause [6]. Generally, venous sinus stenosis leads to the dural sinus's normal contour collapsing or flattening [7]. This case demonstrated classic symptoms of IIH, including visual disturbances and papilledema, with neuroimaging revealing right TSS.

Papilledema refers to optic nerve swelling secondary to raised ICP. It may be seen in conditions such as IIH, intracranial mass lesions, cerebral venous sinus thrombosis, and hydrocephalus. Therefore, neuroimaging with MRI and MR venography is essential to exclude these diagnoses. Lumbar puncture is additionally required to confirm the presence of elevated ICP. Other causes of optic nerve swelling include optic neuritis and ischemic optic neuropathy. In this case, the absence of relative afferent pupillary defect reduced the likelihood of optic neuritis, and the lack of vascular risk factors made ischemic optic neuropathy less likely.

At the time of her first emergency visit, the patient faced socioeconomic constraints, including a lack of insurance, which precluded neuroimaging and more aggressive treatment. Despite initial treatment with acetazolamide and two lumbar punctures, her symptoms returned two weeks later. Acetazolamide is the first-line pharmacological treatment for IIH, and its effect is thought to be mediated by reducing CSF secretion [8]. Lumbar puncture is recommended in patients with papilledema as both a therapeutic and diagnostic tool. Although lumbar puncture can provide temporary relief and help protect vision, its effects are often short-lived because the removed CSF is quickly replenished. Our patient reported symptomatic relief of headaches and did not have signs of low tension or decreased intraocular pressure. Therefore, serial lumbar punctures are not typically recommended as a long-term treatment for IIH [9]. Given the patient’s refractory symptoms and evidence of venous sinus narrowing, she underwent a transverse venous sinus stenting procedure, increasingly recognized as a potential treatment. Following stent placement, the patient denied any recurrent symptoms or complications following the procedure. Venous sinus stenting has been theorized to normalize ICP, thereby relieving compression and reducing the burden on the optic nerve [10]. Surgical risk should be considered when evaluating patients for venous sinus stent placement. These include venous injury or perforation, stent thrombosis, and hemorrhage. To reduce thrombotic complications, patients are typically placed on antiplatelet therapy following the procedure. Optic nerve sheath fenestration (ONSF) is preferred in patients with substantial vision loss without prominent headaches. This procedure directly protects the optic nerve; however, it does not significantly lower ICP. CSF diversion procedures such as lumboperitoneal or ventriculoperitoneal shunts are effective in reducing ICP, but they are associated with high complication rates. Our patient's clinical condition significantly improved after stenting, with notable improvements in visual acuity and field sensitivity as well as a reduction of headaches and papilledema. However, persistent impairments associated with chronic optic nerve damage were found in subsequent perimetry. Thus, highlighting the importance of early recognition and intervention in preventing permanent visual impairment. The present case demonstrates several variables in the assessment and treatment of IIH. In order to evaluate for venous sinus abnormalities, neuroimaging, including MRI and venography, should be performed early in patients with suspected IIH. Neuroimaging is essential to exclude intracranial mass lesions, venous thrombosis, and malignancy. Lumbar puncture serves both diagnostic and therapeutic purposes, with an elevated opening pressure confirming intracranial hypertension and supporting the ophthalmic finding of papilledema. CSF cytology aids in excluding infectious etiologies such as bacterial or viral meningitis. Venous sinus stenting may be effective in refractory or progressing cases, especially those with evident venous sinus stenosis. Finally, even with successful treatment, some patients may experience irreparable visual loss; thus, long-term follow-up is essential.

## Conclusions

This case highlights the importance of thoroughly investigating patients who show signs of pseudotumor cerebri, looking at the possibility of underlying venous sinus abnormalities. Early utilization of neuroimaging, such as MRI and MR venography, can facilitate timely diagnosis and enable early patient-tailored treatment. Although treatments such as acetazolamide and lumbar puncture may offer temporary relief from elevated intracranial pressure, this case highlights the importance of early imaging and prompt, aggressive intervention to prevent symptom recurrence and preserve vision. Venous sinus stenting should be considered as a treatment option, particularly in complex or progressive cases where significant venous narrowing is evident to preserve visual function. As seen in this case, the risk of permanent visual loss highlights the importance of prompt intervention and continuous follow-up after resolution of acute symptoms.
